# Assessment of the relationship between 25-hydroxyvitamin D and albuminuria in type 2 diabetes mellitus

**DOI:** 10.1186/s12902-022-01088-2

**Published:** 2022-07-04

**Authors:** Seyed Alireza Zomorodian, Maryam Shafiee, Zeinab Karimi, Fatemeh Masjedi, Amirhossein Roshanshad

**Affiliations:** 1 Shiraz Nephro-Urology Research Center, Shiraz University of Medical Sciences, Shiraz, Iran; 2 Student Research Committee, Shiraz University of Medical Sciences, Shiraz, Iran

**Keywords:** Albuminuria, Vitamin D, Diabetic nephropathy

## Abstract

**Background:**

Diabetic nephropathy occurs in about one-third of diabetic patients. This health problem is characterized by increased urinary albumin excretion, leading to decreased glomerular filtration rate and renal failure. In this regard, previous investigations have revealed the possibility of a relationship between vitamin D deficiency and diabetic nephropathy. The present study assessed the relationship between vitamin D deficiency and albuminuria in patients with type 2 diabetes.

**Methods:**

This study was conducted with 200 participants with type 2 diabetes mellitus from December 2019 to January 2021. The patients’ 25-hydroxyvitamin D (25OHD) serum level and urinary albumin-to-creatinine ratio (UACR) were measured concurrently. Afterward, the subjects were divided into three groups based on their albuminuria level. Finally, 25OHD serum level and other clinical characteristics were compared among these albuminuria groups, and the relation between albuminuria level and 25OHD was analyzed.

**Results:**

The prevalence of vitamin D deficiency in macroalbuminuric patients (UACR≥300 mg/g) was 61.8%, and in microalbuminuric (30 ≤ UACR< 300 mg/g) and normoalbuminuric groups (UACR< 30 mg/g) was 33.3% and 24%, respectively. Further analysis revealed a significant negative relationship between 25OHD and albuminuria(*r* = − 0.257, *p*-value< 0.001). According to ROC curve analysis, a 25OHD level ≤ 21 ng/ml was considered an optimal cut-off point value for having macroalbuminuria in diabetic patients.

**Conclusions:**

The current study evaluates the relation between vitamin D deficiency and the prevalence of albuminuria in the setting of diabetes. Overall, the prevalence of macroalbuminuria increased when the 25OHD serum level was less than 20 ng/ml.

## Background

Diabetes mellitus is a complex and multi-factorial metabolic syndrome characterized by insulin secretion or insulin activity defects, resulting in hyperglycemia. Insulin resistance and relative insulin deficiency are the main features of diabetes mellitus type 2. Diabetes mellitus has significant macrovascular complications such as coronary heart disease and cerebrovascular accidents and microvascular events, including retinopathy, neuropathy, and nephropathy [1]. Diabetic nephropathy manifests as increased urine albumin excretion, decreased estimated glomerular filtration rate (eGFR), and renal failure. Diabetic nephropathy that eventually results in end-stage renal disease (ESRD) affects around 40 % of patients with diabetes mellitus [2].

Different processes, such as hyperglycemia which induces hemodynamic and metabolic alterations, facilitate the progression of diabetic nephropathy. Also, hemodynamic changes, including activation of the renin-angiotensin-aldosterone and endothelin systems, lead to the release of pro-fibrotic cytokines such as transforming growth factor beta1 (TGF-β1), which increases glomerular and systemic pressure [3, 4]. Research has shown the effect of inflammatory processes and cytokines, including interleukin-1, interleukin-6, interleukin-18, and tumor necrosis factor (TNF), on diabetic nephropathy development [5, 6]. These processes lead to glomerular and tubulointerstitial hypertrophy, thickening of the glomerular basement membrane, extracellular matrix accumulation, and the production of micro-aneurysms and mesangial nodules, resulting in glomerular sclerosis and tubulointerstitial fibrosis [7].

The primary role of vitamin D in calcium-phosphorus hemostasis and skeletal health is well understood. Vitamin D receptors (VDR) are found in almost all tissue like the pancreas, heart, liver, thyroid, brain, and kidney. In this respect, it has been documented that vitamin D, with an inhibitory effect on the renin-angiotensin-aldosterone system (RAAS), has a protective activity on the kidney. In addition, vitamin D promotes the survival of pancreatic beta cells by affecting cytokines and nuclear transcription factors such as NF-κB [8, 9].

Although the significance of vitamin D deficiency in the development of diabetic nephropathy is not known well, several studies demonstrated the protective effect of this vitamin and its analogs on podocytes [10, 11] and their regulatory role in the renin-angiotensin-aldosterone system [12]. Also, some studies have shown the anti-inflammatory and anti-fibrotic effects of vitamin D [13]. While several studies reported vitamin D deficiency as a predictor of diabetic nephropathy [14, 15], some other works have found no association in this regard [16]. Therefore, in the present study, we evaluated the association of vitamin D with albuminuria.

## Methods

### Study subjects and selection

In this cross-sectional study, we used the census method for sampling; individuals with type 2 diabetes mellitus referring to the medical clinics affiliated with Shiraz University of Medical Sciences (Shiraz, Iran) were screened between December of 2019 and January of 2021. Exclusion criteria were 1) age > 80 or < 30 years; 2) glycated hemoglobin (HbA1c) ≥ 8%; 3) estimated glomerular filtration rate (eGFR) < 60 ml/min/1.63 m2 (EPI-equation); 4) uncontrolled blood pressure (BP ≥ 140/90 mmHg); 5) presence of obvious non-diabetic renal disease; 6) uncontrolled hypothyroidism or hyperthyroidism; 7) uncontrolled hyperparathyroidism; 8) malignancy; 9) infectious diseases; 10) rheumatologic diseases; 11) ketoacidosis or hyperosmotic coma in the past three months; 12); body mass index (BMI) ≥ 35 or ≤ 18.5 Kg/m^2^, and 13) taking supplements containing vitamin D or calcium in the preceding four weeks. Also, their medications like anti-hyperglycemic or anti-hypertension agents should not have changed in the past three months.

In this study, we assessed 486 participants with diabetes mellitus type 2. The exclusion criteria eliminated 286 patients, and finally, 200 subjects were evaluated for the analysis. The majority of those who were excluded had a low glomerular filtration rate (eGFR< 60 ml/min/1.73m^2^) or uncontrolled blood sugar (HbA1c ≥ 8%).

Then the enrolled participants were classified according to the UACR as normoalbuminuria (UACR< 30 mg/g), microalbuminuria (30 ≤ UACR< 300 mg/g), or macroalbuminuria (UACR≥300 mg/g). Also, due to variability in albuminuria, at least two of three urinary samples of the participants revealed diagnostic values for micro and macroalbuminuria within the past three to six months for confirmation of persistent albuminuria, which is recommended by the American Diabetes Association (ADA).

The ethics committee of Shiraz University of Medical Sciences authorized this study (IR.SUMS.MED.REC.1399.109), and all participants submitted written informed consent.

Data related to demographic characteristics, duration of diabetes, and personal medical histories were collected by prepared physicians. BMI was computed by dividing weight (kg) by height squared (m^2^) taken from the participants. After resting for 5 min, blood pressure was taken in the sitting poison at the left brachial artery with a manual sphygmomanometer. Afterward, a resting venous blood sample (5 ml) was taken by drawing blood into spray-dried K2EDTA tubes from an antecubital vein. The concentration of 25OHD in serum samples was then determined using an available ELISA kit (Infinitum Biotech, El Cajon, CA, USA, sensitivity: 1.69 ng/ml). Finally, a urine container was given to the patient to collect a urine sample for evaluating UACR. Other laboratory data were collected from the past month’s medical records.

### Statistical analysis

Data were checked using the Shapiro-Wilk normality test. One-way analysis of variance (ANOVA) was applied to compare data with normal distribution, and the Kruskal-Wallis test was applied if the data were not normally distributed. A post hoc test (Bonferroni correction) was carried out to compare significance within each variable. The bivariate Spearman correlation coefficient showed the correlations between continuous quantitative variables.

The chi-squared test was used to evaluate if there is a relationship between two categorical variables. Also, the receiver operating characteristic (ROC) curve was used to establish the ideal vitamin D cut-off value for predicting diabetic nephropathy severity with the highest sensitivity and specificity. In this study, the best cut-off point was the 25OHD concentration at the related criterion of the greatest Youden Index (J) from the Roc curve using the MedCalc software (MedCalc 20 Software bvba, Ostend, Belgium). Demographic and experimental data were expressed using means and standard deviations. For all statistical analyses, the statistical significance level was evaluated using a *p*-value< 0.05. Moreover, GraphPad Prism for Windows was used to analyze the data (Version 8.0, GraphPad Software Inc. La Jolla, California, USA).

## Results

The study population included 123 females and 77 men with a mean age of 58.75 ± 9.37 years and a mean diabetes duration of 10.99 ± 8.03 years. About 34% of participants were affected by diabetic retinopathy, and 71.2% were affected by hypertension. According to the UACR, participants were classified as normoalbuminuria (UACR< 30 mg/g), microalbuminuria (30 ≤ UACR< 300 mg/g), or macroalbuminuria (UACR≥300 mg/g). Of 200 subjects, 100 were classified as normoalbuminuria, 66 as microalbuminuria, and 34 as macroalbuminuria.

### Clinical characteristics of different albuminuria groups

Table 1 presents the clinical characteristics of the study population classified into three different albuminuria groups. HbA1c, triglyceride, low-density lipoprotein cholesterol (LDL-C), parathyroid hormone (PTH), sodium, potassium, calcium, and phosphorous blood levels did not differ significantly across groups. There was a statistically significant difference in high-density lipoprotein cholesterol (HDL-C), uric acid, and 25OHD serum levels. Also, subjects in the microalbuminuria and macroalbuminuria groups were taking angiotensin-converting enzyme inhibitors or angiotensin receptor blockers (ACEIs/ARBs) significantly more than in the control albuminuria.

**Table 1:** The baseline characteristics of the participants according to albuminuria

Characteristics ^a^	Normoalbuminuria (UACR< 30 mg/g)	Microalbuminuria (30 ≤ UACR< 300 mg/g)	Macroalbuminuria (UACR> 300 mg/g)	*p*-value
Age (yr)	58.87 ± 9.36	59.17 ± 9.75	57.59 ± 8.80	0.718
Male/female	29/71	32/34	16/18	0.022
Diabetes duration (yr)	11.17 ± 7.85	12.00 ± 8.88	8.54 ± 6.05	0.220
Hypertension (%)	64.3%	73.9%	78.3%	0.427
ACEIs/ARBs use (%)	72.7%	92%	95.8%	0.008
BMI (kg/m^2^)	27.25 ± 4.71	28.05 ± 4.11	28.81 ± 2.66	0.347
HbA1c^c^ (%)	6.47 ± 0.96	6.90 ± 0.93	7.05 ± 0.70	0.196
BUN (mg/dl)	14.40 ± 4.73	15.58 ± 5.47	17.30 ± 4.74	0.016
Creatinine (mg/dl)	0.95 ± 0.17	1.00 ± .018	1.01 ± 0.22	0.084
eGFR (ml/min/1.73m^2^)	74.17 ± 13.66	72.28 ± 18.28	72.28 ± 8.28	0.595
Sodium (mmol/lit)	139.76 ± 2.95	139.54 ± 2.61	140.06 ± 3.91	0.753
Potassium (mEq/lit)	4.38 ± 0.48	4.41 ± 0.40	4.36 ± 0.41	0.904
Serum uric acid (mg/dl)	4.59 ± 1.28	5.30 ± 1.42	5.79 ± 1.44	0.003
Calcium (mg/dl)	9.39 ± 0.47	9.53 ± 0.56	9.56 ± 0.45	0.213
Phosphorus (mg/dl)	3.84 ± 0.66	3.84 ± 0.57	4.01 ± 0.69	0.529
PTH (ng/lit)	39.92 ± 19.11	39.30 ± 27.29	46.62 ± 24.64	0.540
TSH (mIU/lit)	2.93 ± 2.15	2.51 ± 1.58	2.24 ± 1.38	0.233
Triglycerides (mg/dl)	152.16 ± 83.20	166.48 ± 97.45	193.16 ± 95.89	0.096
Total cholesterol (mg/dl)	158.56 ± 40.17	149.79 ± 43.37	154.67 ± 30.36	0.445
LDL-C (mg/dl)	83.10 ± 33.81	82.80 ± 32.47	84.16 ± 27.37	0.982
HDL-C (mg/dl)	47.28 ± 14.42	41.23 ± 9.31	40.07 ± 3.61	0.007
UACR^b^ (mg/g)	4.45 ± 11	71.50 ± 73.75	530.00 ± 592.00	< 0.001
25OHD (ng/ml)	29.99 ± 13.69	27.57 ± 13.39	18.30 ± 8.12	< 0.001
Vitamin D Deficiency(%)	24.0%	33.3%	61.8%	< 0.001

^a^ Data were reported as mean ± standard deviation or number

^b^ UACR was reported as median ± Interquartile range

*Abbreviations*: *ACEIs/ARBs* Angiotensin-converting enzyme inhibitors or angiotensin receptor blockers, *BMI* Body mass index, *HbA1c* Glycated hemoglobin, *BUN* Blood urea nitrogen, *eGFR* Estimated glomerular filtration rate, *PTH* Parathyroid hormone, *TSH* Thyroid-stimulating hormone, *LDL-C* Low-density lipoprotein cholesterol, *HDL-C* High-density lipoprotein cholesterol, *UACR* Urinary albumin-to-creatinine ratio, *25OHD* 25-hydroxyvitamin D

### The level of 25-hydroxyvitamin D at various stages of albuminuria

In patients with macroalbuminuria, the mean blood level of 25OHD was considerably lower than in patients with normoalbuminuria (18.31 ± 8.12 ng/ml vs. 29.99 ± 13.69 ng/ml). In addition, the 25OHD level showed a significant difference between diabetic patients with micro and macroalbuminuria. Vitamin D deficiency rate (25OHD < 20 ng/ml) was 24% among patients with normoalbuminuria, 33.3% in the microalbuminuria group, and 61.8% in those with macroalbuminuria. Furthermore, the severe vitamin D deficiency rate (25OHD < 12 ng/ml) was significantly higher in macroalbuminuria patients than in those with normal or microalbuminuria.

### The correlation of 25-hydroxyvitamin D and albuminuria

**Fig. 1:**
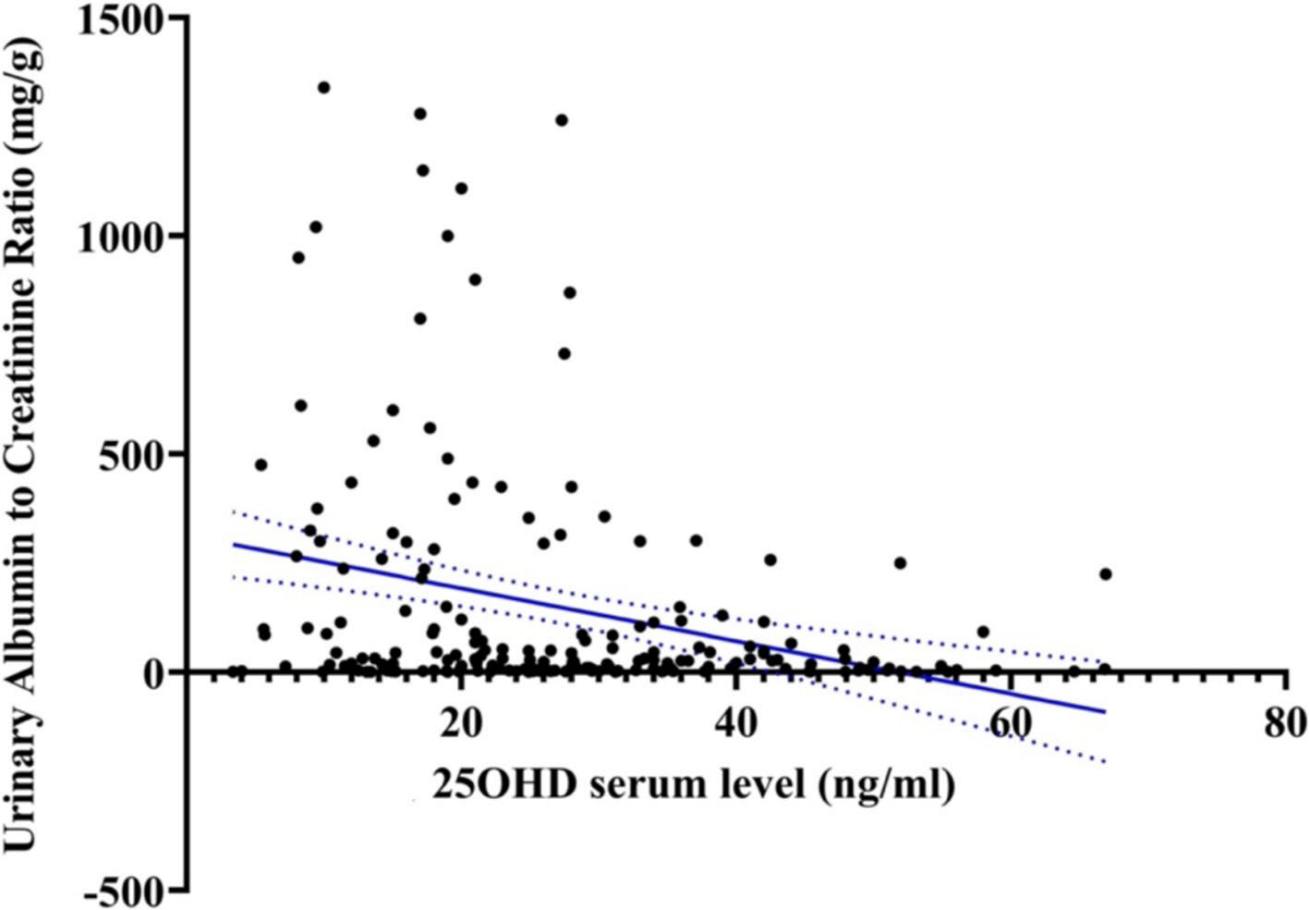
The correlation analysis of 25OHD and albuminuria; Spearman correlation between 25OHD serum level and UACR in the study population (*n =* 200) shows a significant reverse relation between them (*r* = − 0.257; *p*-value< 0.001)

The relationship between 25OHD serum levels and UACR in the study population is shown in Figure 1. A strong negative correlation between serum level of 25OHD and severity of albuminuria was identified using the Spearman correlation test (*r* = − 0.257, *p*-value< 0.001).

### ROC curve interpretation

**Fig. 2:**
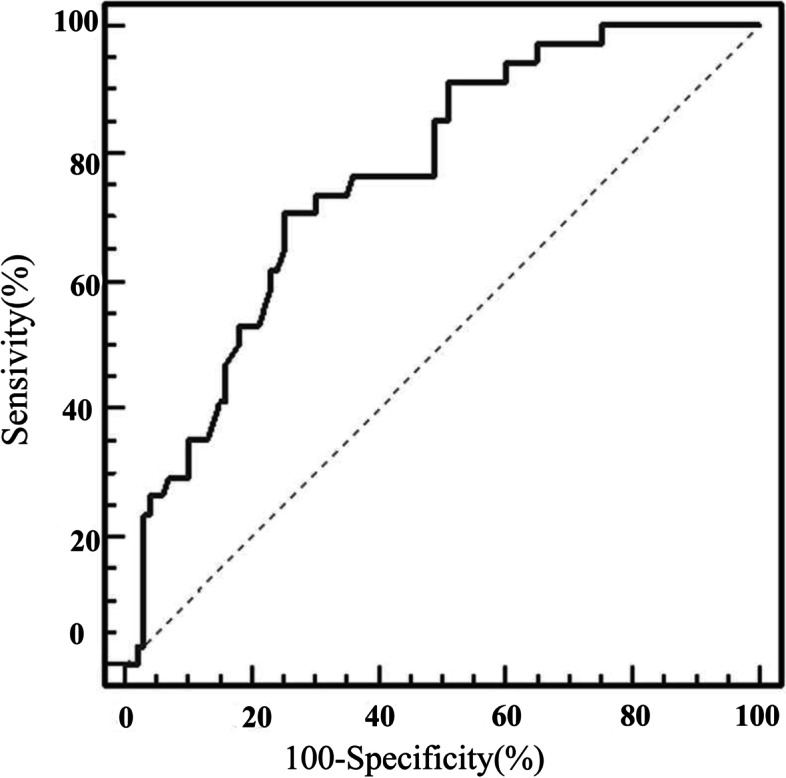
ROC curve for determining the optimal 25OHD (vitamin D) cut-off point in predicting macroalbuminuria

Figure 2 illustrates the ROC curve for identifying the optimal 25OHD cut-off point associated with macroalbuminuria. The area under the curve (AUC) was 0.763 (95% CI = 0.682 to 0.832), with 70.6% sensitivity and 75% specificity. The ideal vitamin D cut-off point (J) for macroalbuminuria was ≤21 ng/ml.

### The association of vitamin D deficiency and the severity of albuminuria

In the chi-squared test, the probability of the association of vitamin D deficiency and severity of albuminuria was assessed. The 25OHD level was categorized into two groups of vitamin D deficiency (25OHD < 20 ng/ml) and vitamin D sufficiency (25OHD ≥ 20). The frequency of vitamin D deficiency was compared between normoalbuminuric and microalbuminuric patients, and also between normoalbuminuric and macroalbuminuric cases. The results showed that people with vitamin D deficiency were more likely to be microalbuminuric than normoalbuminuric, with an odds ratio (OR) of 1.58 (95% CI = 0.796 to 3.149). In addition, people with vitamin D deficiency had a 5.11 times higher chance of developing macroalbuminuria than patients with sufficient vitamin D levels (OR = 5.11, 95% CI = 2.23 to 11.73).

## Discussion

The present study discovered that 25-hydroxyvitamin D serum levels were inversely correlated with albuminuria as an indicator of diabetic nephropathy in patients with type 2 diabetes mellitus, and a 25OHD serum level ≤ 21 ng/ml could have a predictive value for macroalbuminuria.

Vitamin D has renal protective effects, including reducing pro-fibrotic growth factors and inflammatory cytokines such as TGF-β. Furthermore, it may suppress the renin-angiotensin-aldosterone system (RAAS), the main renal injury player in diabetic nephropathy [17]. According to previous research, vitamin D deficiency is more common in patients with considerable albuminuria than those without albuminuria [14, 18]. They observed that macroalbuminuric and microalbuminuric patients had a higher incidence of vitamin D deficiency. Another study demonstrated that 25OHD deficiency (25OHD < 20 ng/ml) was independently correlated with elevated UACR as an indicator of diabetic nephropathy [19]. In addition, multiple clinical trials have shown that vitamin D administration can slow the progression of diabetic nephropathy by lowering albuminuria levels [20, 21].

In our study, the 25OHD level was considerably lower in the macroalbuminuria group than in the normo or microalbuminuria groups. In contrast, there was no statistically significant difference between microalbuminuric and normoalbuminuric subjects (*p*-value = 0.264).

We adjusted all parameters that affect albuminuria in this investigation; it was even done for patients’ medication. All participants with albuminuria greater than 30 mg/g had taken the highest tolerated dose of ACEIs/ARBs during the previous three months. It is known that ACEIs/ARBs have an albuminuria-reducing effect [22].

Overall, a better glycemic control reduces the risks of microvascular complications such as primary retinopathy or nephropathy in patients with type 2 diabetes because every 1% reduction in HbA1c is correlated with better outcomes over time [23, 24]. Hence, we decided to enroll diabetic patients with better glycemic control than those who participated in the Xie et al. study (HbA1c 8% vs. HbA1c 11%) [14].

 In another study, Hong SH et al. assessed the relation between serum 25OHD and diabetic nephropathy [19]. However, the researchers did not consider uncontrolled diabetes and chronic kidney disease, although albuminuria is associated with glomerular filtration rate and lower glomerular filtration along with the progression of chronic kidney disease related to rising albuminuria [25]. Accordingly, we eliminated all patients with eGFR < 60 ml/min/1.73 m2 to lower the influence of low glomerular filtration on albuminuria.

Further analysis revealed a significant association between vitamin D deficiency and macroalbuminuric diabetic nephropathy, particularly when 25OHD is less than 20 ng/ml. The cut-off value for predicting macroalbuminuria using 25OHD was 21 ng/ml. This value is close to the 20 ng/ml cut-off level used by the Endocrine Society Task Force on Vitamin D to define vitamin D deficiency [26]. Similarly, R. He et al. presented the cut-off value of 25OHD for predicting sight-threatening diabetic retinopathy at 15.57 ng/ml [27]. Due to the prevalence of 22 to 62% of diabetic retinopathy in albuminuric diabetic nephropathy [28, 29]; probably, 25OHD deficiency could also be one of the factors that affect the progression of diabetic nephropathy.

In the present study, the frequency of vitamin D deficiency in normo or microalbuminuric patients was approximately equal to the normal population but significantly higher in macroalbuminuric patients [30]. The trend of kidney changes in diabetic patients is glomerular hyperfiltration, mesangial expansion, inflammation, and finally, glomerulosclerosis and tubulointerstitial fibrosis [31]. According to study results and progression of inflammation and fibrosis in macroalbuminuria, vitamin D seems to have anti-inflammatory and anti-fibrotic effects [32, 33]; it probably has a more association with the development of macroalbuminuria. Macroalbuminuria often progresses to ESRD and accompanies a worse cardiovascular prognosis [34]. Cardiovascular events are the most common cause of death in diabetic patients. In this regard, albuminuria increases cardiovascular events risk in diabetes [35]. Therefore, the progression of diabetic nephropathy to ESRD and related cardiovascular events could be declined by correcting the serum level of vitamin D and reducing albuminuria.

There are some limitations in this research that should be considered appropriately. First, nutrition, sun exposure duration, and season of vitamin D detection all affected 25OHD levels, which have not been considered in this study. Also, the finding of macroalbuminuric patients who had the study’s criteria was the study’s limitation; because most of the patients with macroalbuminuria had eGFR< 60 ml/min/1.73m^2^. Since no cross-sectional study has investigated long-term data on vitamin D levels and albuminuria, more research is needed to determine the precise relationship and underlying mechanism between low 25OHD and albuminuria in diabetic patients.

## Conclusions

In conclusion, 25OHD serum level was inversely associated with albuminuria. Correcting vitamin D deficiency may lower macroalbuminuria and cardiovascular events in diabetic patients. However, more prospective cohort studies and clinical trials on a large scale are required to clarify the causal relationship between vitamin D and albuminuria.

## Data Availability

The data that support the findings of this study are available from the research deputy of Shiraz University of Medical Sciences, but restrictions apply to the availability of these data, which were used under license for the current study, and so are not publicly available. Data are, however, available from the authors upon reasonable request and with permission of the research deputy of Shiraz University of Medical Sciences.

## Abbreviations

25OHD: 25-hydroxyvitamin D
eGFR: Estimated Glomerular Filtration Rate
UACR: Urinary Albumin-to-Creatinine Ratio
ESRD: End-Stage Renal Disease
TGF-β1: Transforming Growth Factor beta1
TNF: Tumor Necrosis Factor
RAAS: Renin-Angiotensin-Aldosterone System
